# Coverage assessment survey following trachoma mass drug administration (MDA) in six districts of Oromia, Western Ethiopia, 2017

**DOI:** 10.1371/journal.pntd.0007924

**Published:** 2019-12-16

**Authors:** Tariku Tesfaye Bekuma, Getu Mosisa Kebebew, Zelalem Desalegn Waktole, Jote Markos Cafo, Desalegn Wirtu, Solomon Gaddisa

**Affiliations:** 1 Department of Public Health, Institute of Health Sciences, Wollega University, Nekemte, Ethiopia; 2 School of Nursing and Midwifery, Institute of Health Sciences, Wollega University, Nekemte, Ethiopia; 3 Light for the World International, Addis Ababa, Ethiopia; RTI International, UNITED STATES

## Abstract

**Background:**

Trachoma is a contagious infection of the eye by specific strains of the bacteria *Chlamydia trachomatis*. It is the leading cause of blindness worldwide. Mass drug administration (MDA) with azithromycin is a cornerstone of World Health Organization (WHO)’s global effort to eliminate trachoma by 2020. This coverage survey was aimed to assess trachoma post-mass drug administration (MDA) coverage among six selected districts of East Wollega, Horo Guduru Wollega, and West Shewa zones in2017.

**Methods:**

A community based cross-sectional coverage survey was conducted. The sample size was calculated automatically using Coverage Survey Builder (CSB) tool in microsoft excel. Thirty segments were selected per each selected districts of the three zones. A separate Results Entry Form for each district surveyed was completed, saved and uploaded directly into the online Coverage Survey Analysis Tool to estimate the surveycoverage and the program reach along with the corresponding 95% confidence limits and design effects. EPI-INFO 7.0 and SPSS version 20 was used for further analysis of survey data.

**Result:**

A total of 1,747 households were surveyed, out of which 10,700 individuals were interviewed. Most respondents (95.1%) stated that they heard about trachoma MDA and most of them replied that they got the information from health workers. Program reach ranged between 89.5% in Jimma Geneti district and 94.8% in Dirre Hinchini district. The most common mentioned reasons for not having taken azithromycin included not knowing about the campaign, fear of side effects and being absent during the MDA campaign.

**Conclusion:**

In this survey, four of the six districts met the target threshold (i.e. 80%) for effective coverage; Ambo rural and Jimma Geneti did not meet the target threshold.Therefore, programmatic improvements should be made for the future campaign to reach the expected thresholds while the campaign in four of the six districts should be encouraged.

## Author summary

Trachoma is a contagious infection of the eye and the leading cause of blindness worldwide. Mass drug administration (MDA) is one of the WHO initiated a global program to eliminate trachoma by 2020. Mass drug administration is a strategy used to administer medicines to the entire at-risk population of an area, most commonly a district using a campaign-style approach. This coverage survey was aimed to assess knowledge and source of information about the previous trachoma mass drug administration campaign, its coverage level, and the reason for not taking the drug. It highlighted the previous challenges, existing gaps and explained differences in coverages reported within the respective districts. Therefore, future MDA rounds will be able to take into account these findings and reach target populations that might have been missed during previous rounds of MDA. For this survey, it was found that mass drug administration coverage was lower than reported one by the districts, except for Haro Limu and Boneya Boshe. The difference was as small as 2% in Haro Limu district to as high as 20% in Boneya Boshe district. Therefore, in the districts with lower MDA coverage, improvements should be made for the future campaign to reach the expected thresholds.

## Introduction

Trachoma is a contagious infection of the eye caused by specific strains of the bacteria *Chlamydia trachomatis*. Active infection often begins during infancy or childhood and can become chronic. The bacteria are spread by direct contact with eye and nose discharges from infected individuals, by contact with fomites (i.e., inanimate objects that carry infectious agents, such as towels or washcloths) or by eye-seeking flies (particularly *Musca sorbens*). It is the leading infectious cause of blindness and is endemic in 53 countries. An estimated 325 million people live in areas where they can be exposed to trachoma, and more than 7 million suffer from trichiasis, the final painful stage of this eye disease.[1, 2, 3]

A total of 165.1 million people lived in districts in which the TF prevalence in children aged 1–9 years was ≥5% during 2017; of these, 89% (146.3 million) were in WHO’s African Region, and 42% (69.8 million) were in Ethiopia.[4] Blinding trachoma can be eliminated by implementing an integrated package of interventions—the so-called “SAFE strategy” which stands for: surgery for trachomatous trichiasis; antibiotic treatment to clear ocular *C*. *trachomatis* infection; facial cleanliness to reduce transmission of ocular *C*. *trachomatis*; and environmental improvement, particularly improved access to water and sanitation. A total of 83.5 million people globally received azithromycin for elimination of trachoma in 2017; 60% of the doses distributed in 2017 were distributed in Ethiopia, the country with the largest population at risk.[2, 4]

There are more than 75 million people living in trachoma-endemic areas in Ethiopia. The national prevalence of active trachoma either Trachomatous Inflammation Follicular (TF) or Trachomatous Inflammation Intense (TI) for children aged 1–9 years is 40.1%. The highest prevalence is found in Amhara (62.6%), Oromia (41.3%), and SNNP (33.2%), Ethiopia’s largest regional states. In major urban areas, active trachoma prevalence is very low. The national prevalence of Trachomatous Trichiasis (TT) for the age group 15 and above is 3.1% [5, 6]. More than 200 districts in Ethiopia are now working to eliminate trachoma through a partnership of the Federal Ministry of Health, Regional Health Bureaus and many international and local development partners.[7]

Mass drug administration (MDA) to the entire population is used to prevent, control, or eliminate neglected tropical diseases (NTDs) like trachoma, whereby drugs (azithromycin for trachoma) are administered periodically—using a campaign style approach—to the entire at-risk population of an area, most commonly a district.

Coverage matters for trachoma MDAs: the higher the prevalence of infection, the more important it is to achieve high coverage. WHO recommends that at least 80% of the target population should be reached with MDA. Country programmes routinely report treatment coverage by subtracting the number of doses of azithromycin left in stock after MDAs from the MDA target population, or by summing the reports from drug distributors. While both of these methods are better than doing nothing, it is important to check the accuracy of such routinely reported coverage figures, as they are subject to manipulation and error.

Drug coverage estimates from population-based surveys may increase our understanding of factors affecting the effectiveness of MDA [8, 9, 10]. Survey findings have provided valuable information on the existing gaps, to projects targeting prevention and elimination of the disease. Future MDA rounds will be able to take into account the findings of this survey and will enable trachoma control programmes to reach target populations that might have been missed during previous rounds of MDA. Since 2015, Oromia’s Regional Health Bureau (ORHB), in partnership with Light for the World, conducted trachoma MDA in 42 districts of East Wollega (17 districts), Horoguduru Wollega (9 districts), West Shewa (15 districts) and west Wollega (1 district) zones.

The aim of the survey reported here was to assess post-MDA coverage in East Wollega, Horoguduru Wollega, and West Shewa zones, Ethiopia, in 2017.

## Methods

### Survey area and period

**Fig 1:**
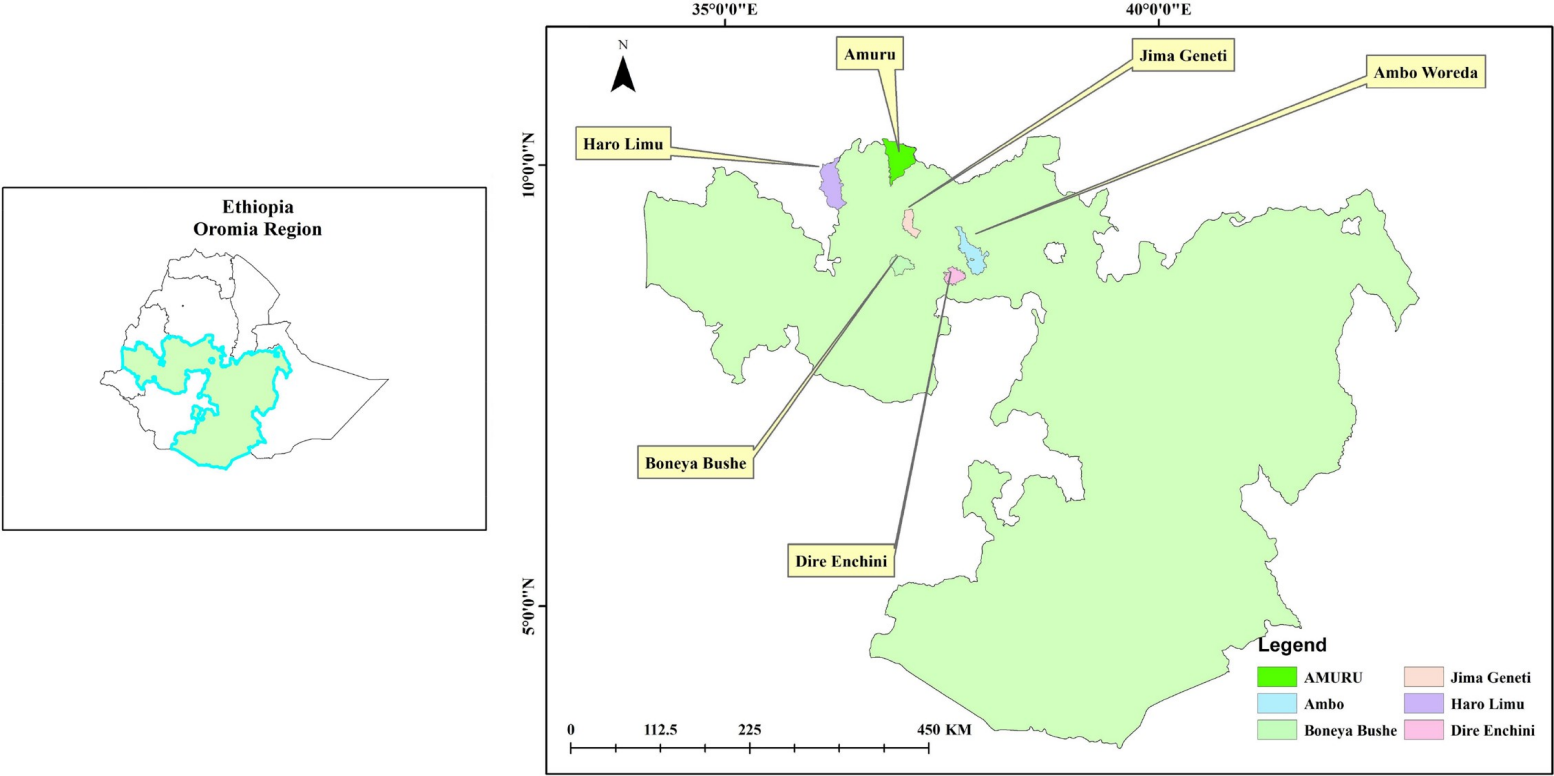
Map showing the six survey districts of Oromia Regional state, Ethiopia, 2017.

This coverage survey was conducted from August 1–16, 2017, in purposively selected trachoma intervention districts of East Wollega, Horo Guduru Wollega, and West Shewa zones of Oromia Regional State. These zones are trachoma-endemic and a first and second round of trachoma MDA was completed in December 2015 and March 2017, respectively. The target beneficiaries of the MDA are over 4 million people in the 42 trachoma intervention districts (Fig 1).

### Survey design

Community-based cross-sectional coverage survey was implemented.

### Survey population

All residents/population living in the selected districts was surveyed population.

### Eligible population

Everybody living in the survey area (district)based on drug-specific eligibility criteria.

#### Inclusion criteria

Everybody living in the survey area, where recent Trachoma MDA was administered was considered to be included in the study.

#### Exclusion criteria

Those households which were closed during the survey data collection period. In addition, non-resident individuals who came to visit relatives from other location were excluded.

### Sample size

The sample size, n, was determined using the formula for a single population proportion:

$$n=\frac{{\left(Z_{\alpha /2}\right)}^{2}x\;p\;x\;q\;x\;\mathrm{DEFF}}{d^{2}}$$

Where: α is level of confidence;

p is the proportion of the population who is expected to have swallowed the drug is considered to be 50%. The expected coverage sample size will increase as the reported coverage approaches 50%, which ensures that the sample size is sufficient to meet study objectives;
q is (1-p);
Z is standard normal distribution curve value for 95% CI which is 1.96 (where α = 0.05);
d is tolerable margin of error, i.e. 5% (0.05); and
DEFF is the design effect, a measure that reflects the degree to which respondents in the same subunit are likely to be similar in terms of the information provided in response to; we used the suggested default of 4

A sample size of 1,537 was estimated; including a 10% non-response margin, the final sample size was estimated at 1,691 individuals.

### Sampling techniques

The World Health Organization (WHO) guideline for implementation of coverage surveys for preventive chemotherapy was used.[11] In a first step, the number of households (HHs) in each district was estimated by dividing their respective total population to the estimated households’ size of Oromia (i.e. 4.8 per HH). Out of the 42 districts’ survey population, six districts were selected purposively based on MDA coverage report history. Accordingly, two districts each East Wollega (Boneya Boshe and Haro Limu), Horo Guduru Wollega (Jimma Geneti and Amuru) and West Shewa (Ambo Rural and Dirre Hinchini) were included in the survey. The list of study kebeles in the respective districts were used. On arrival, the selected kebeles mapped and geographically divided into segments of approximately 50 households and proportionally allocated number of segments for the kebele was selected. With the help of local guide, path through the segments that passes by each household in the segments was identified. Finally, the segments from the respective kebeles were selected at random and a fixed proportion of its households are selected for the survey interview. Because the fresh listing of households requires extra time and cost, a combination oftherandom route and quota sampling technique were employed.

### Data collection tools

The survey questionnaire was adopted from the WHO guideline for implementation of coverage surveys which comprises questions related to knowledge and source of information about trachoma; factors affecting trachoma drug uptake and overall trachoma MDA coverage. It also includes questions on participation in the MDA, which type of medication the person took, as well as reasons for not taking the drugs. The questionnaire was prepared in English and translated to the local language, Afan Oromo. For most questions, multiple possible answers were provided.

### Survey team

The survey team included district level staffs/health professionals who were not involved in the MDA campaign in sampled districts. Each survey team was typically comprised of 7 individuals: 5 interviewers, one supervisor and one driver, all fluent in Afan Oromo. A-three-day training was organized to traing the team on the survey, including on sampling techniques, informed consent, and interview process.

### Data quality control

Practical training was given to the survey team and a pre-test was conducted in Wayu Tuka district, a site that was selected outside of the survey sites. During the data collection, supervisors have watched over the data collectors on site and every evening checked the data for accuracy, consistency, and completeness. The filled out questionnaires was collected and handled properly. The data was double-eneterd into the “Results Entry Form” by two separate individuals to address any discrepancies between the two, in order to minimize data entry errors.

### Data processing and analysis

To calculate the MDA coverage, the CSB provided Results Entry Form was used. The total number of people interviewed, the number of people who reported being offered the drug and the number of people who reported swallowing the drug was entered for each selected segment. A separate Results Entry Form for each district surveyed was then completed, saved and uploaded directly into the online Coverage Survey Analysis Tool. The program finally returned the estimate for the survey coverage and the program reach along with the corresponding 95% confidence limits and design effects. In addition, the data was entered using EPI-INFO 7.0 and analyzed using SPSS software Version 20. Descriptive analysis and summary statistics on MDA coverage was produced for the overall population, by age and gender. The association between the factors and the uptake of trachoma drugs was analyzed using Pearson’s Chi-square test. Moreover, factors affecting trachoma MDA coverage were identified using Logistic regression.

### Survey variables

The dependent variable is trachoma MDA coverage. independent variables include socio-demographic characteristics such as sex, age, religion, educational status; knowledge about MDA; knowledge about trachoma; source of information about trachoma MDA; type of drug taken; drug side effects; drug shortage, and others.

### Operational definition

The survey area is the 42 districts in which MDA was conducted and preventive chemotherapy coverage is tabulated and reported. A segment is defined as a grouping of approximately 50 households (HHs) from within the initially sampled district; used for sampling efficiency. A HH is defined as a group of people who eat and live together. The eligible population is defined as the population targeted for MDA treatment, based on drug-specific eligibility criteria. The survey population is defined as the population for which an estimate of preventive chemotherapy coverage is desired. A permanent resident is defined as individuals who live in the area for at least six months. MDA is defined as the administration of either azithromycin or Tetracycline Eye Ointment (TEO) to whole populations irrespective of disease status. Coverage is defined as the number of people treated (with either azithromycin or TEO) divided by the total number of residents. Survey Coverage is defined as the estimation of the total number of individuals surveyed divided to the total number of individuals surveyed who were identified as having ingested the drug.

### Ethical considerations

The Wollega University Ethical Review Board provided ethical clearance and approval for the study; leaders of each selected district for the coverage survey were made aware of the survey in advance of the team’s visit with a letter written by Wollega University. During this sensitization visit (or phone call) with the local leaders, the representative from the survey team shared the purpose of the coverage survey and also discussed the optimal day of the week and time of day for the survey team to visit in order to find members of the survey population at home. Written consent was obtained from participants over 18 years of age. If the person was <18 years old, written informed consent was taken from the head of HH or responsible adult (guardian). In addition to consent by the representing adult.verbal assent was required for each child.

## Results

### Socio-demographic characteristics

A total of 1,747 HHs were surveyed, ranging from 249 in Boneya Boshe district and 396 in Ambo rural district; the HH comprised a total population of 10,700 people. A large proportion of heads of HHs and indeed individuals surveyed could not read and write across all districts (Table 1).

**Table 1:** Educational status of respondents and HH heads among six districts of East Wollega, Horo Guduru Wollega and West Shewa zones survey population, 2017.

	**Districts**					
**Educational status of the respondent**	**Boneya Boshe** **N = 249**	**Haro Limu** **N = 287**	**Jimma Geneti** **N = 252**	**Amuru** **N = 329**	**Dirre Hinchini** **N = 234**	**Ambo Rural** **N = 396**
**Cannot read and write**	42.97%	43.90%	30.95%	51.06%	30.34%	46.21%
**Can read and write**	4.02%	13.24%	1.59%	30.70%	0.85%	2.27%
**1 to 4**	24.50%	12.89%	12.30%	5.17%	20.09%	16.41%
**5 to 8**	19.28%	19.16%	27.78%	6.69%	21.79%	21.21%
**9 to 10**	6.83%	6.97%	17.46%	3.34%	15.38%	9.85%
**11 to 12**	0.80%	0.70%	3.57%	1.22%	5.13%	2.02%
**College+**	1.61%	3.14%	6.35%	1.82%	6.41%	2.02%
**Total**	100%	100%	100%	100%	100%	100%
**Educational status of HH head**	**N = 249**	**N = 287**	**N = 252**	**N = 329**	**N = 234**	**N = 396**
**Cannot read and write**	43.37%	39.37%	30.56%	47.72%	26.07%	37.88%
**Can read and write**	4.42%	14.29%	1.98%	34.04%	3.42%	4.04%
**1 to 4**	24.90%	13.24%	9.52%	6.08%	14.96%	18.43%
**5 to 8**	17.27%	23.34%	25.79%	6.38%	23.93%	23.48%
**9 to 10**	6.83%	4.53%	16.67%	2.74%	18.38%	10.35%
**11 to 12**	0.80%	2.09%	5.95%	1.22%	3.42%	2.78%
**College+**	2.41%	3.14%	9.52%	1.82%	9.83%	3.03%
**Total**	100%	100%	100%	100%	100%	100%

The highest surveyed populations were within 35–39 years of age group in Boneya Boshe, Haro Limu and Dirre Hinchini districts, within 30–34 years of age group in Jimma Geneti and Ambo Rural districts and ≥60 years of age group in Amuru district. (Table 2)

**Table 2:** Age distribution of respondents in six districts of East Wollega, Horo Guduru Wollega and West Shewa zones of Oromia, 2017.

	Districts					
Age of the respondents (years)	Boneya Boshe N = 249	Haro Limu N = 287	Jimma Geneti N = 252	Amuru N = 329	Dirre Hinchini N = 234	Ambo Rural N = 396
**15–19**	2.01%	4.18%	6.75%	4.26%	5.98%	6.82%
**20–24**	5.22%	4.53%	6.35%	3.95%	6.41%	10.86%
**25–29**	16.47%	11.50%	16.67%	8.51%	11.97%	13.89%
**30–34**	15.26%	19.16%	19.44%	15.81%	15.81%	15.91%
**35–39**	21.29%	21.95%	14.68%	11.25%	18.80%	12.37%
**40–44**	13.65%	15.68%	10.71%	12.46%	8.55%	10.86%
**45–49**	8.84%	9.06%	7.94%	7.60%	3.85%	6.57%
**50–54**	6.83%	4.88%	6.35%	11.25%	7.69%	6.57%
**55–59**	4.02%	3.48%	2.78%	5.17%	3.42%	4.04%
**≥60**	6.43%	5.57%	8.33%	19.76%	17.52%	12.12%
**Total**	100%	100%	100%	100%	100%	100%

### Knowledge of respondents about trachoma

Out of the surveyed HHs, 1,282 (73.4%) had received information about trachoma, with health workers and radio the major source of information about trachoma in all surveyed districts; banners and posters were the most minor sources of information (Table 3).

**Table 3:** Source of nformation about trachoma as reported by respondents, in six districts of East Wollega, Horo Guduru Wollega, and West Shewa zones of Oromia, 2017.

	Districts					
Source of information	Boneya Boshe N = 157	Haro Limu N = 184	Jimma Geneti N = 224	Amuru N = 290	Dirre Hinchini N = 159	Ambo Rural N = 268
**TV**	9.55%	3.26%	11.16%	1.38%	20.13%	10.45%
**Radio**	57.96%	48.91%	25.89%	16.55%	24.53%	30.97%
**School**	12.10%	26.63%	19.64%	13.45%	14.47%	10.82%
**Teachers**	12.10%	8.15%	8.04%	3.79%	16.98%	14.18%
**Posters**	0.00%	1.09%	0.45%	0.00%	1.26%	2.99%
**Banner**	1.27%	1.09%	0.89%	0.00%	0.63%	0.37%
**Leaflet**	1.91%	1.09%	0.00%	2.07%	0.00%	1.87%
**Health workers**	75.80%	75.00%	95.09%	88.28%	55.97%	58.58%
**Public announcement**	2.55%	2.17%	16.52%	5.86%	1.89%	2.99%
**Religion place**	3.82%	2.72%	4.46%	0.00%	5.03%	5.22%
**Family member**	7.64%	8.70%	5.36%	0.00%	6.92%	8.21%
**Friend**	6.37%	5.43%	1.34%	0.34%	11.95%	19.78%
Others*	5.73%	2.17%	0.89%	0.34%	1.88%	3.35%
**Total**	100%	100%	100%	100%	100%	100%

*Gare leader, social and local training

From those who thought that trachoma is transmitted from person to person, the fly was the most frequently mentioned transmission modality in all districts except for Haro Limu where not getting eye treatment was mentioned as main transmission modality. Other transmission modalities such as dust, smoke, air, looking at the infected person and reading in the sun were reported in four districts. Respondents’ awareness about trachoma revealed that discharge from the eye was the most commonly mentioned sign and symptom of trachoma except Boneya Boshe and Haro Limu districts. Concerning trachoma prevention methods of trachoma, except for Dirre Hinchini and Ambo Rural districts, keeping personal hygiene followed by environmental hygiene were the most frequently mentioned approaches (Table 4).

**Table 4:** Respondents awareness regarding trachoma among six districts of East Wollega, Horo Guduru Wollega and West Shewa zones survey population, 2017.

Trachoma transmission modality	Boneya Boshe	Haro Limu	Jimma Geneti	Amuru	Dirre Hinchini		Ambo Rural
	N = 130	N = 153	N = 176	N = 196	N = 86		N = 167
**Not washing face**	42 (32.31%)	83 (54.25%)	124 (70.45%)	111 (56.63%)	12 (13.95%)		33 (19.76%)
**Not getting eye treatment**	71 (54.62%)	97 (63.40%)	49 (27.84%)	68 (34.69%)	2 (2.33%)		6 (3.59%)
**Sharing utensils**	69 (53.08%)	67 (43.79%)	23 (13.07%)	55 (28.06%)	40 (46.51%)		55 (32.93%)
**Sharing washing materials**	58 (44.62%)	90 (58.82%)	36 (20.45%)	69 (35.20%)	17 (19.77%)		18 (10.78%)
**Fly**	96 (73.85%)	96 (62.75%)	97 (55.11%)	81 (41.33%)	57 (66.28%)		139 (83.23%)
**Others**	2 (1.54%)	0 (0.00%)	2 (1.14%)	0 (0.00%)	3 (3.49%)		3 (1.80%)
**Sign and symptom of trachoma**	**N = 157**	**N = 184**	**N = 224**	**N = 290**		**N = 159**	**N = 268**
**Red eye**	108 (68.79%)	132 (71.74%)	115 (51.34%)	135 (46.55%)		38 (23.90%)	89 (33.21%)
**Eye discharge**	80 (50.96%)	113 (61.41%)	123 (54.91%)	143 (49.31%)		107 (67.30%)	168 (62.69%)
**Eyelid swelling**	63 (40.13%)	82 (44.57%)	48 (21.43%)	94 (32.41%)		13 (8.18%)	31 (11.57%)
**Itching of eye and eyelid**	90 (57.32%)	115 (62.50%)	97 (43.30%)	129 (44.48%)		64 (40.25%)	81 (30.22%)
**Photophobia**	44 (28.03%)	71 (38.59%)	54 (24.11%)	64 (22.07%)		38 (23.90%)	59 (22.01%)
**Don't know**	6 (3.82%)	8 (4.35%)	14 (6.25%)	13 (4.48%)		18 (11.32%)	39 (14.55%)
**Trachoma prevention methods**	**N = 249**	**N = 287**	**N = 252**	**N = 329**		**N = 234**	**N = 396**
**Health Education**	48 (19.28%)	54 (18.82%)	42 (16.67%)	53 (16.11%)		5 (2.14%)	8 (2.02%)
**Washing face**	88 (35.34%))	105 (36.59%)	86 (34.13%)	108 (32.83%)		47 (20.09%)	107 (27.02%)
**Not sharing washing materials**	54 (21.69%)	86 (29.97%)	31 (12.30%)	86 (26.14%)		6 (2.56%)	23 (5.81%)
**Personal hygiene**	126 (50.60%)	153 (53.31%)	189 (75.00%)	224 (68.09%)		98 (41.88%)	187 (47.22%)
**Environmental hygiene**	104 (41.77%)	132 (45.99%)	131 (51.98%)	137 (41.64%)		60 (25.64%)	128 (32.32%)
**Taking medication**	50 (20.08%)	73 (25.44%)	43 (17.06%0	44 (13.37%)		92 (39.32%)	135 (34.09%)
**Other**	4 (1.61%)	7 (2.44%)	5 (1.98%)	5 (1.52%)		9 (3.85%)	23 (5.81%)

### Information about MDA

Of survey respondents, 1,662 (95.1%) mentioned that they had heard about trachoma MDA and most of them replied that they got the information from health workers; other main sources of information included radio and schools respectively, while posters and banners were the most minor sources of information for trachoma MDA. (Table 5).

**Table 5:** Source of information about Trachoma MDA campaign in six districts of East Wollega, Horo Guduru and West Shewa zones of Oromia, 2017.

Source of MDA Information	Boneya Boshe	Haro Limu	Jimma Geneti	Amuru	Dirre Hinchini	Ambo Rural
	N = 232	N = 270	N = 238	N = 310	N = 231	N = 381
**TV**	3.88%	5.93%	5.88%	0.32%	4.76%	1.57%
**Radio**	34.48%	44.44%	13.45%	2.90%	2.16%	5.25%
**School**	13.79%	32.96%	21.01%	15.16%	7.36%	4.72%
**Teachers**	4.74%	5.93%	8.40%	3.55%	0.43%	3.94%
**Poster**	0.00%	0.74%	0.84%	0.97%	0.43%	0.26%
**Banner**	0.43%	1.48%	0.84%	0.00%	0.00%	0.00%
**Leaflets**	2.16%	5.19%	0.42%	0.32%	0.00%	0.52%
**Health workers**	84.05%	73.70%	92.44%	98.39%	80.95%	85.56%
**Public announcement**	4.74%	1.11%	21.43%	6.77%	19.91%	14.96%
**Religious place**	12.93%	4.81%	4.62%	0.65%	9.09%	6.56%
**Family member**	13.36%	8.52%	2.94%	0.00%	3.90%	4.72%
**Friends**	6.47%	4.07%	1.26%	0.00%	3.90%	5.77%
**Others**	13.79%	8.15%	0.00%	0.65%	3.90%	9.71%
**Don’t know**	0.86%	2.22%	0.00%	0.00%	0.00%	0.00%

Note: Denominator is total respondent swho heard about MDA

Among the total surveyed population, the proportion of male interviewees was higher than females except for Boneya Boshe and Ambo rural districts. In Boneya Boshe, Haro Limu and Amuru districts, the highest surveyed population was aged between 10–19 years while the age under 10 years was the highest in the rest three districts. Across all the districts, people aged greater than 60 years were the fewest of all age groups (Table 6).

**Table 6:** Sex and age distribution of the interviewed population among East Wollega, Horo Guduru Wollega and West Shewa zones, 2017.

Variable	Categories	Boneya Boshe N = 1685	Haro Limu N = 1947	Jimma Geneti N = 1357	Amuru N = 1979	Dirre Hinchini N = 1920	Ambo Rural N = 1812
**Sex**	Male	48.8%	52.5%	50.4%	51.2%	51.2%	49.8%
	Female	51.2%	47.5%	49.6%	48.8%	48.8%	50.2%
**Total**		100%	100%	100%	100%	100%	100%
**Age group**	<10	27.4%	30.4%	30.2%	28.9%	30.5%	32.9%
	10–19	33.8%	32.8%	29.3%	29.7%	29.4%	29.0%
	20–29	14.4%	12.8%	13.8%	12.8%	13.2%	12.7%
	30–39	13.2%	13.0%	13.1%	10.2%	10.8%	10.6%
	40–49	6.7%	6.4%	7.5%	7.7%	6.1%	6.2%
	50–59	2.9%	2.7%	3.1%	5.1%	4.0%	4.2%
	60–69	1.2%	1.3%	1.7%	3.5%	3.1%	2.3%
	>69	0.4%	0.5%	1.3%	2.3%	2.9%	2.1%
**Total**		100%	100%	100%	100%	100%	100%

### Mass drug administration (MDA) coverage

#### Survey coverage

The survey coverage for the respective Districts was calculated using the formula:

$$\mathrm{Survey}\;\mathrm{coverage}=\frac{Number\;of\;“yes”\;responses\;to\;having\;swallowed\;the\;drug}{Total\;number\;of\;people\;interviewed}$$

Accordingly, the survey coverage for trachoma MDA for Ambo rural district was 83%, Dirre Hinchinni 88%, Haro Limu 90%, Jimma Geneti 81%, Amuru 86%, and Boneya Boshe 90%.

Because the survey coverage only slightly greater than the target coverage threshold (<10 percentage points) for the four districts namely Ambo Rural, Dirre Hinchini, Amuru and Jimma Geneti, the data were entered into the online Coverage Analysis Tool to determine the 1-sided lower confidence bound. Thus, the online tool returns confidence bound of 82.7% (79.2%-86.3%, DEFF = 3.6) for Ambo Rural, 88.5 (85%-92.1%, DEFF = 5.2) for Dirre Hinchini, 86.4% (81.5%-91.3%, DEFF = 9) for Amuru, and 80.5% (73.5%-87.5%, DEFF = 9.3) for Jimma Geneti.

For Haro Limu and Boneya Boshe districts the CSB result, the survey coverage of 90% is taken as it is without online analysis since it is ≥10% of the target threshold.

#### Program reach

To determine how well the programme was able to reach the population, the coverage of the programme reach was calculated as follows:

$$\mathrm{Program}\;\mathrm{reach}=\frac{Number\;of\;“yes”\;responses\;to\;having\;been\;offered\;the\;drug}{Total\;number\;of\;people\;interviewed}=$$

Accordingly, program reach was calculated and those <90% except Boneya Boshe and Haro Limu were entered into an online analysis tool—93.8% (90–97.5%, DEFF = 10.5) for Amuru, 89.5% (83.5–95.6%, DEFF = 11.6) for Jimma Geneti, 92.9% (90.5–95.4% DEFF = 3.5) for Ambo Rural and 94.8% (91.3–98.2%, DEFF = 10.2 for Dirre Hinchini).

Out of the total interviewed individuals in the selected districts, 9,897 (92.5%) people reported that they were offered to participate in azithromycin MDA, with minimum and maximumin program reach being 89.5% in Jimma Geneti and 94.8% in Dirre Hinchini, respectively. Out of respondents who were offered to participate in the MDA, 9,273 (93.69%) people reportedly swallowed the drug, with survey coverage ranging from 80.5% in Jimma Geneti to 90% in Boneya Boshe and Haro Limu (Table 7).

**Table 7:** Proportion of Program Reach and Survey Coverage of MDA Distribution among six districts of East Wollega, Horo Guduru Wollega and West Shewa Zones, Oromia, 2017.

Districts	Program Reach	Survey coverage	Online Analysis Tool result	
			Program reach	Survey coverage
**Boneya Boshe**	91%	90%	-	-
**Haro Limu**	92%	90%	-	-
**Amuru**	94%	86%	93.8% (90–97.5%, DEFF = 10.5)	86.4% (81.5–91.3%, DEFF = 9)
**Jimma Geneti**	90%	81%	89.5% (83.5–95.6%, DEFF = 11.6)	80.5% (73.5–87.5%, DEFF = 9.3)
**Ambo Rural**	93%	83%	92.9% (90.5–95.4%, DEFF = 3.5)	82.7% (79.2–86.3%, DEFF = 3.6)
**Dire Hinchini**	95%	88%	94.8% (91.3–98.2%, DEFF = 10.2)	88.5% (85–92.1%, DEFF = 5.2)

**Fig 2:**
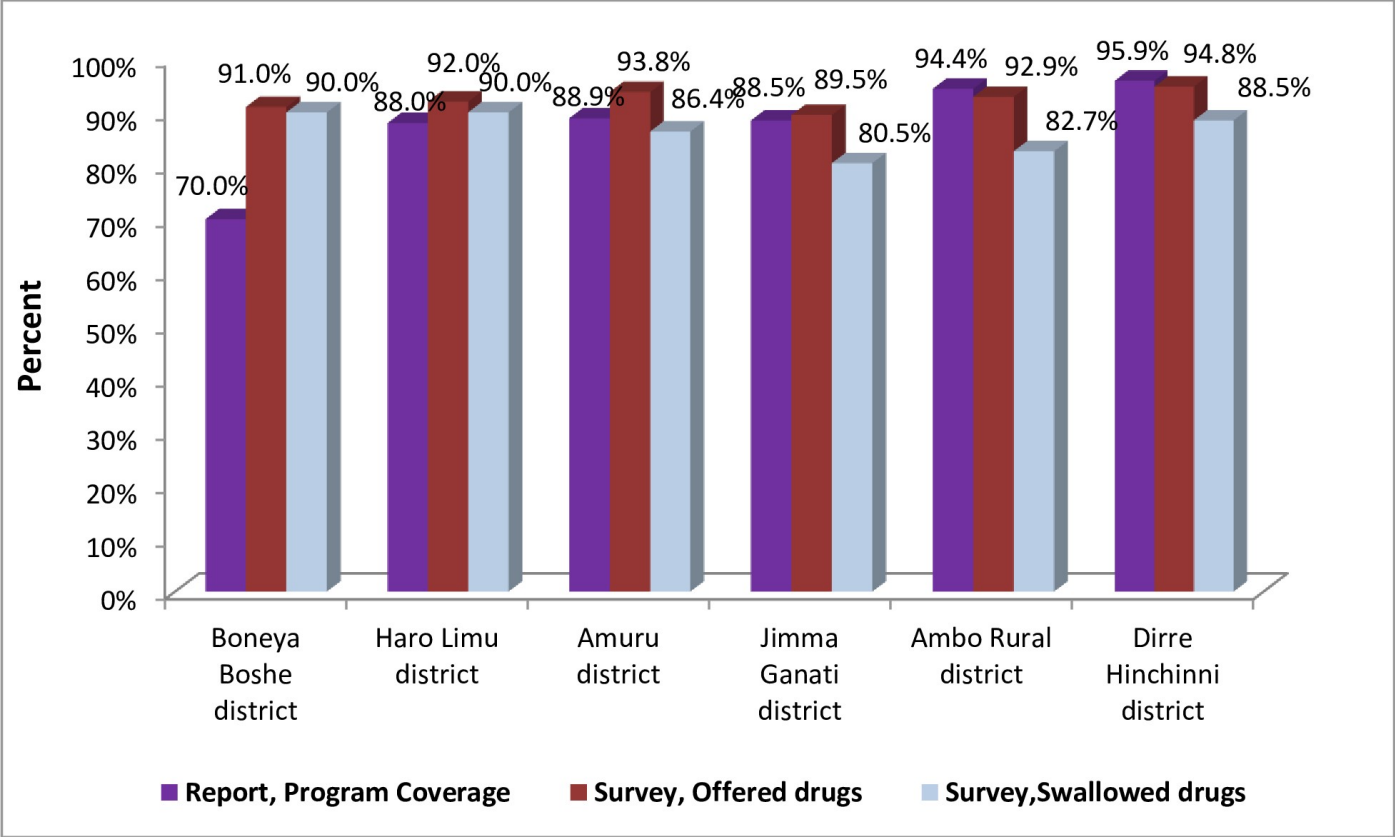
Comparison of MDA coverage from routine report and survey in six districts of East Wollega, Horroguduru Wollega and West Shewa zones, Oromia, 2017.

MDA coverage from the survey wss lower than reported coverage, except for Haro Limu and Boneya Boshe districts. The difference was as small as 2% in Haro Limu district to as high as 20% in Boneya Boshe district (Fig 2).

**Fig 3:**
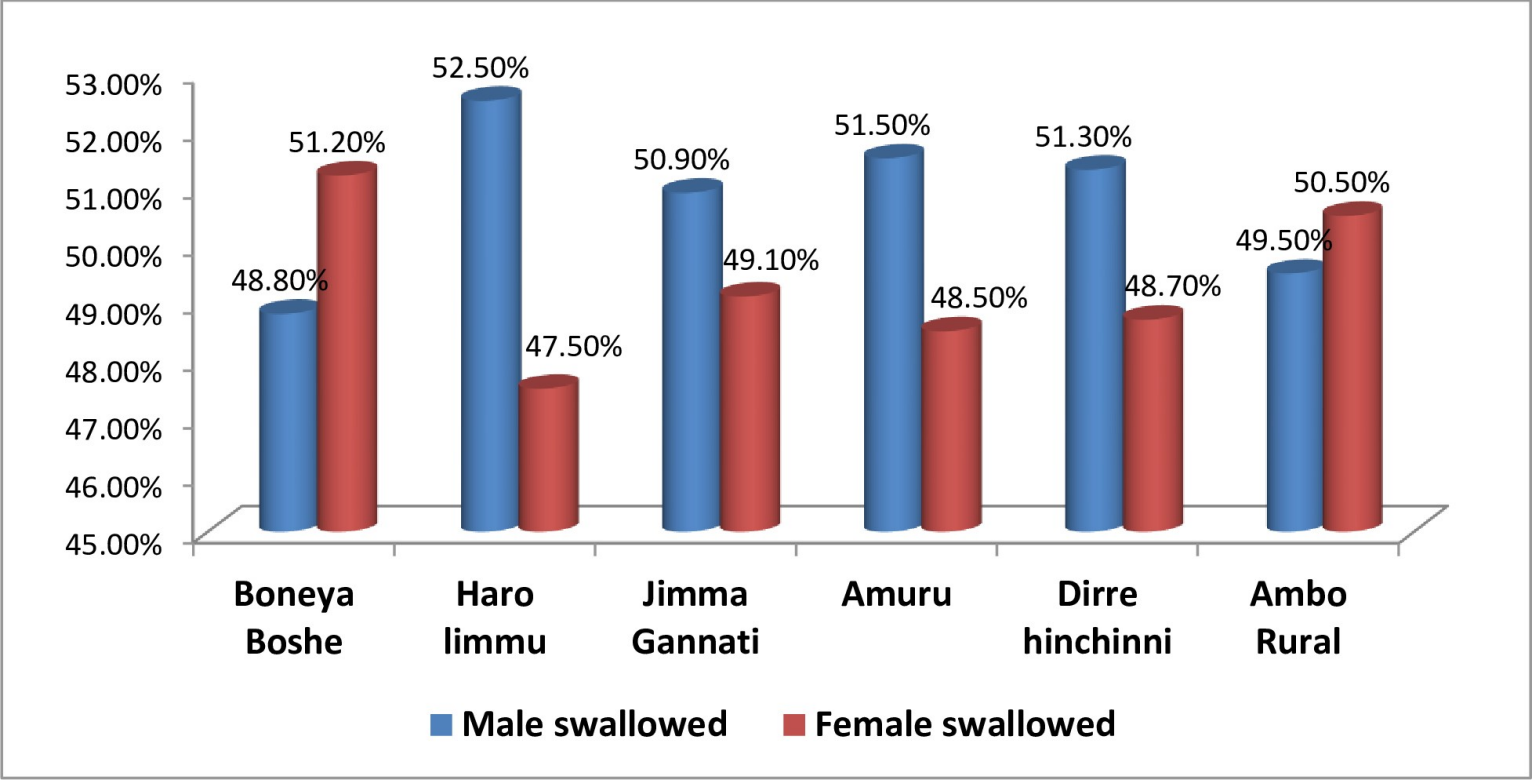
Proportion of respondents swallowed drugs by sex, mass drug administration for Trachoma coverage survey in six districts in East Wollega, Horroguduru Wollega, and West Shewa zones, 2017.

**Fig 4:**
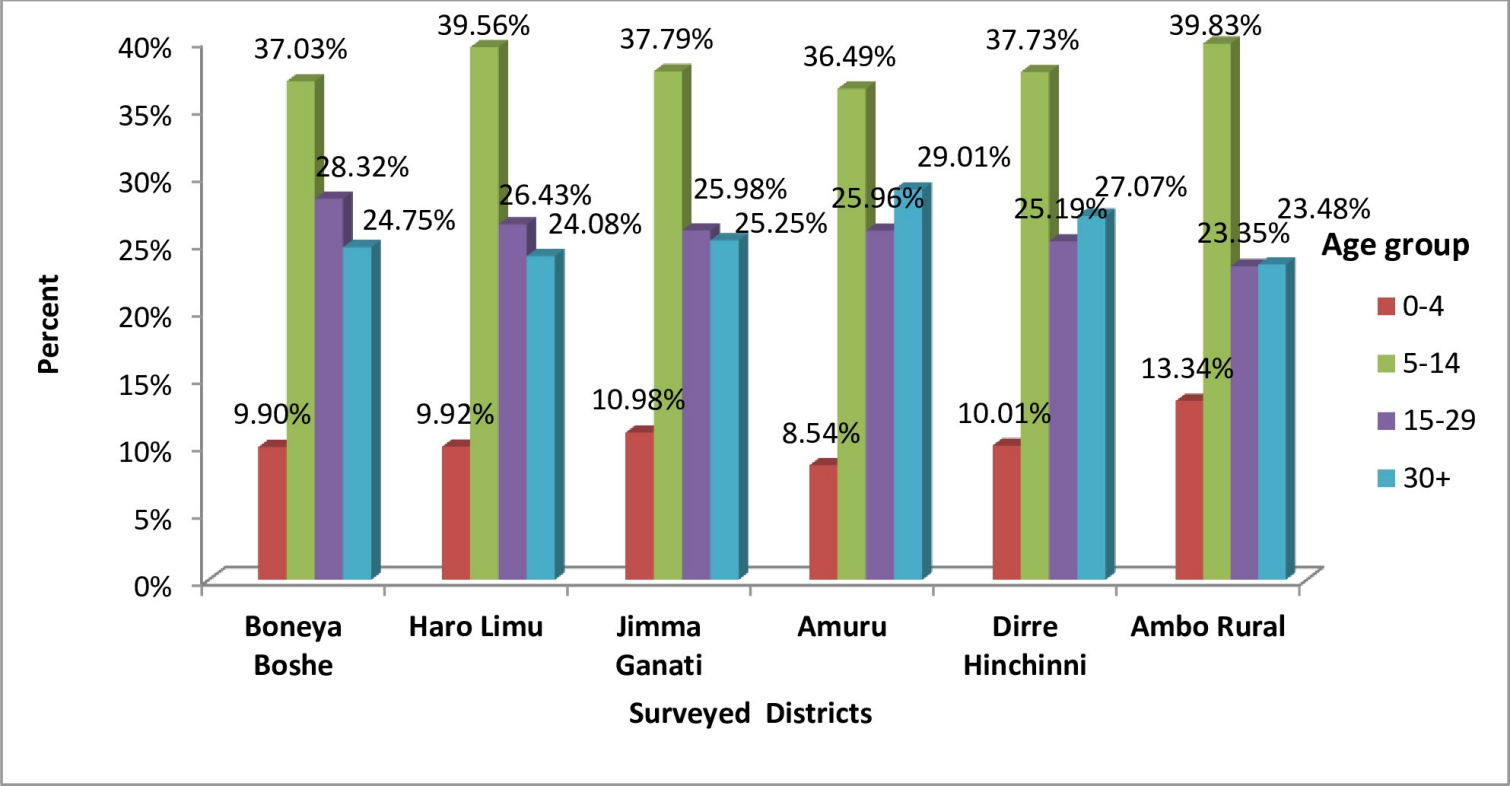
Proportion of respondents swallowed drugs by age group, mass drug administration for Trachoma coverage survey in six districts in East Wollega, Horroguduru Wollega and West Shewa zones, 2017.

Of the total population who swallowed the drug in Boneya Boshe district 48.8% were male and 51.2% were female, in Haro Limu52.5% were male and 47.5% were female, in Jimma Geneti 50.9% were male and 49.1% were female, in Amuru51.5%were male and 48.5% female, in Dirre Hinchini 51.3% were male and 48.7% were female, in Ambo Rural, 49.5% were male and 50.5% were female(Fig 3). Regarding age of respondents who swallowed the drug, the result showed that those aged between 5–14 were the highest group who swallowed the drug across all the surveyed districts (Fig 4).

### Reasons for not offered and swallowed the MDA drugs

Among people who did not get an offer of participating in Azithromycin MDA, the most frequently mentioned reasons were did not hear about the campaign followed by absent during the campaign except Boneya Boshe and Dirre Hinchini districts in which absent during campaign and underage were the most mentioned respectively. Breastfeeding and pregnancy were mentioned as a reason only in the three districts namely, Amuru, Dirre Hinchini and Ambo Rural. On the other hand, drug run out was mentioned as a reason in the other three districts; Boneya Boshe, Haro Limu, and Jimma Geneti(Table 8).

**Table 8:** Reason for not offered MDA drugs in six districts in the three zones of Oromia, Ethiopia, 2017.

Reason for not offered MDA drug	Boneya Boshe N = 148	Haro Limu N = 162	Jimma Geneti N = 142	Amuru N = 123	Dirre Hinchini N = 100	Ambo Rural N = 128
**Underage**	1.35%	9.88%	4.23%	17.89%	30.00%	14.06%
**Pregnant**	0.00%	0.00%	0.00%	3.25%	2.00%	3.13%
**Breast feeding**	0.00%	0.00%	0.00%	0.81%	1.00%	0.00%
**Sick**	0.00%	1.23%	2.11%	0.81%	3.00%	1.56%
**Absent**	54.73%	6.79%	13.38%	29.27%	16.00%	28.13%
**Didn't hear**	39.86%	46.30%	38.73%	41.46%	24.00%	38.28%
**Drug runout**	1.35%	20.37%	0.70%	0.00%	0.00%	0.00%
**Nobody came**	0.00%	9.88%	33.10%	0.00%	2.00%	10.16%
**Others**	2.70%	5.56%	7.75%	6.50%	22.00%	4.69%
**Total**	**100%**	**100%**	**100%**	**100%**	**100%**	**100%**

Note: Denominator is people who did not get an offer of MDA drugs

**Fig 5:**
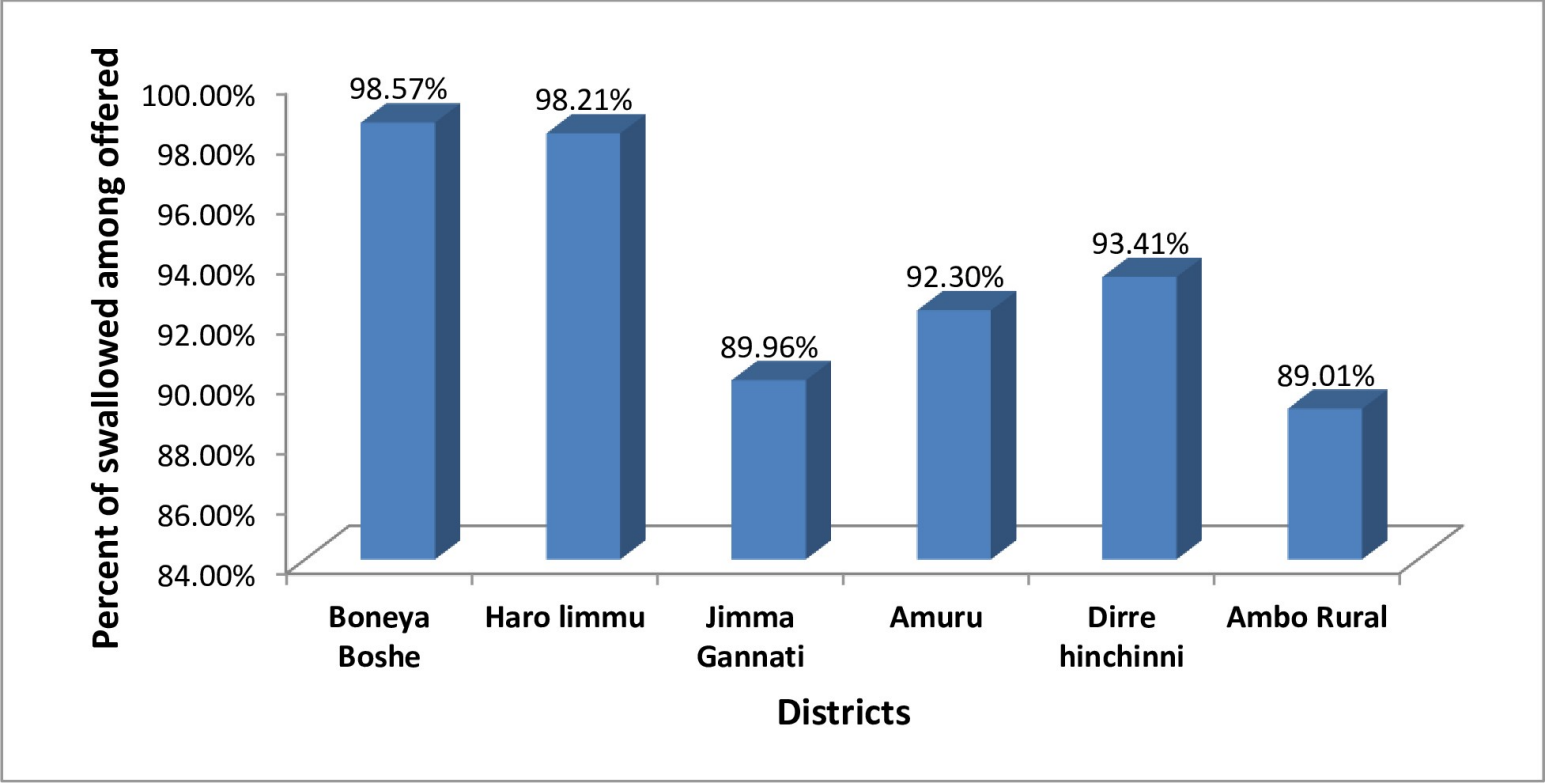
Proportion of people swallowed the drug among those get an offer of the drug in six districts of East Wollega, Horroguduru Wollega, and West Shewa zones, 2017.

Regarding coverage compliance among those who were offered MDA drugs, the survey indicated that the highest and lowest compliance was recorded in Boneya Boshe district (98.57%) and Ambo Rural district (89.01%), respectively (Fig 5).

Among people who did not swallow the drug, the most frequently mentioned reasons were fear of side-effects in Boneya Boshe and Jimma Geneti while absence during the MDA campaign was mentioned as a reason for not swallowing in all the six districts. Being underage was a reason mentioned in Dirre Hinchini, Boneya Boshe, Haro Limu and Ambo Rural while drug distributor did not come was mentioned only in Jimma Geneti and Amuru districts (Table 9).

**Table 9:** Reasons for not swallowing MDA drugs in six districts of East Wollega, West Shewa and Horo Guduru Zone Wollega, Oromia 2017.

Districts						
Reasons for not swallowed	BoneyaBoshe	Haro Limu	JimmaGeneti	Amuru	Dirre Hinchini	Ambo Rural
	n = 22	n = 33	n = 122	n = 146	n = 120	n = 186
**Underage**	18.18%	15.15%	3.28%	4.11%	20.83%	12.37%
**Medicine does not work**	0.00%	0.00%	0.00%	2.05%	9.17%	1.08%
**Fear of side-effects**	40.91%	9.09%	40.16%	0.68%	15.83%	19.35%
**Rumors**	0.00%	3.03%	4.92%	1.37%	1.67%	0.54%
**Too many tablets**	0.00%	0.00%	0.82%	0.00%	0.00%	0.00%
**Too old**	4.55%	3.03%	0.00%	0.68%	0.83%	0.00%
**Pregnant**	4.55%	3.03%	3.28%	0.68%	5.00%	2.69%
**Breastfeeding**	0.00%	0.00%	0.82%	0.68%	1.67%	0.00%
**Too sick**	9.09%	0.00%	4.10%	1.37%	1.67%	1.61%
**Absent**	22.73%	30.30%	17.21%	37.67%	20.83%	35.48%
**Not heard about program**	0.00%	0.00%	3.28%	17.81%	20.00%	22.58%
**Drugs finished**	0.00%	0.00%	0.82%	0.00%	0.83%	1.61%
**Drug distributors did not come**	0.00%	0.00%	18.03%	32.19%	0.00%	0.54%
**Is health**	0.00%	0.00%	0.00%	32.19%	1.67%	2.15%
**Others**	0.00%	36.36%	3.28%	0.68%	0.00%	0.00%
**Total**	100.00	100.00	100.00	100.00	100.00	100.00

Note:—The denominator is subjects who did not swallow the MDA drugs.

### Side-effects

**Fig 6:**
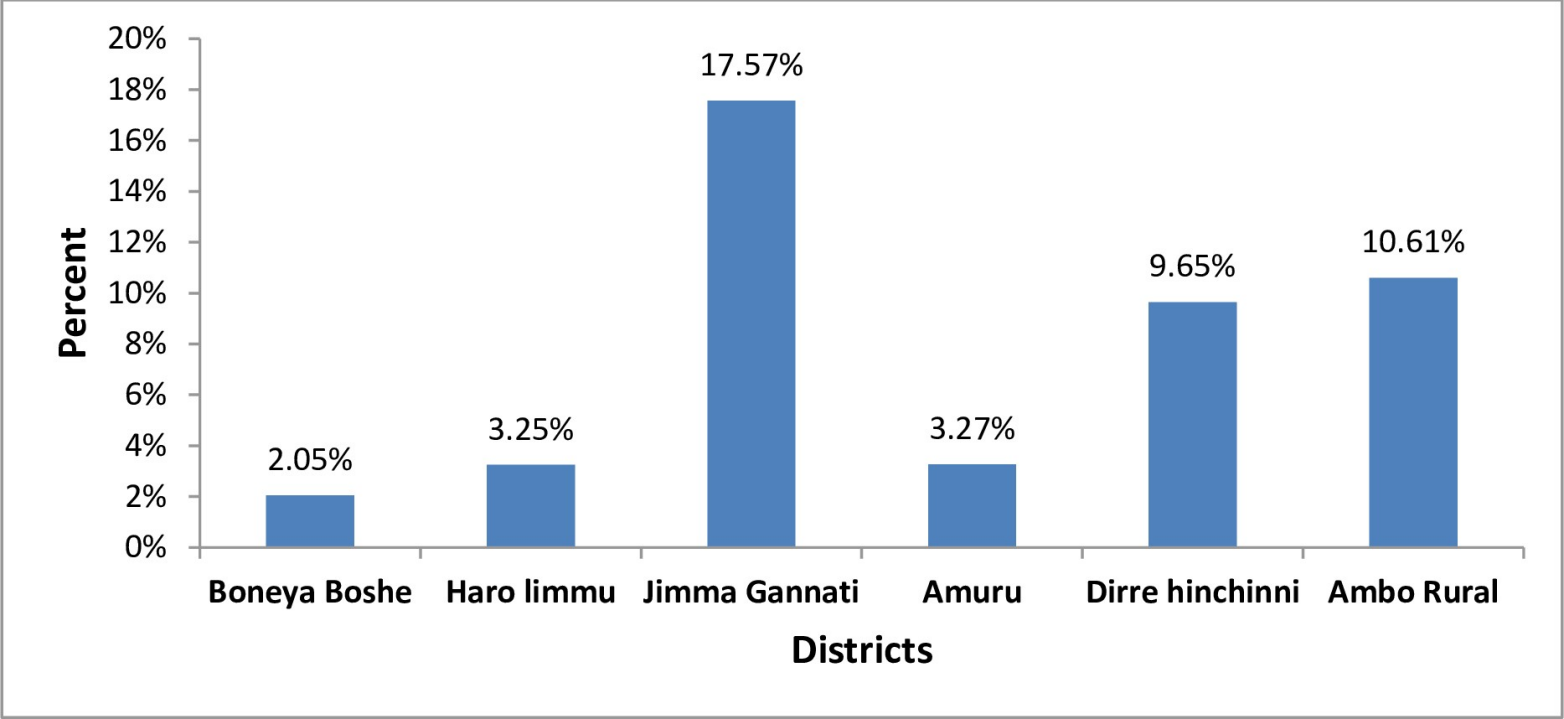
Proportion of respondents who experienced side effects after swallowing MDA drugs, mass drug administration for trachoma coverage survey in six districts of East Wollega, West Shewa and Horroguduru Wollega Zones, Oromia 2017.

Out of the individuals who swallowed the drug, 659 (7.1%) stated to have experienced side-effects. The occurrence of self-reported MDA side-effects varied between districts, from as low as 2.05% in Boneya Boshe to as high as 17.57% in Jimma Geneti (Fig 6). Among those who had side-effects, diarrhea was the most mentioned side-effect in five districts, except in Haro Limu where headache was the commonly mentioned side-effect (Table 10).

**Table 10:** Type of side effects of MDA, mass drug administration for trachoma coverage survey of six districts in East Wollega, West Shewa and Horo Guduru Zone, Oromia 2017.

Type of side effects	Boneya Boshe	Haro Limu	Jimma Geneti	Amuru	Dirre Hinchini	Ambo Rural
	n = 31	n = 57	n = 192	n = 56	n = 164	n = 159
**Vomiting**	6 (19.35%)	4 (7.01%)	46 (23.96%)	10 (17.85%)	13 (7.92%)	26 (16.35%)
**Skin rash**	5 (16.13%)	5 (8.77%)	2 (1.04%)	1 (1.78%)	2 (1.22%)	0 (0.0%)
**Diarrhea**	7 (22.58%)	5 (8.77%)	85 (44.27%)	14 (25%)	59 (35.97%)	82 (51.57%)
**Fever**	1 (3.22%)	4 (7.01%)	6 (3.12%)	4 (7.14%)	2 (1.22%)	1 (0.62%)
**Headache**	4 (12.9%)	19 (33.3%)	12 (6.25%)	6 (10.71%)	59 (35.97%)	16 (10.06%)
**Wheezing**	0 (0.0%)	1 (1.75%)	0 (0.0%)	0 (0.0%)	0 (0.0%)	0 (0.0%)
**Joint pain**	3 (9.67%)	3 (5.26%)	3 (1.56%)	0 (0.0%)	6 (3.65%)	10 (6.28%)
**Dizziness**	3 (9.67%)	14 (24.56%)	7 (3.64%)	13 (23.21%)	7 (4.26%)	9 (5.66%)
**Malaise**	1 (3.22%)	0 (0.0%)	17 (8.85%)	5 (8.9%)	10 (6.09%)	11 (6.9%)
**Photophobia**	1 (3.22%)	2 (3.51%)	3 (1.56%)	0 (0.0%)	2 (1.22%)	1 (0.62%)
**Others**	0 (0.0%)	0 (0.0%)	11 (5.73%)	3 (5.35%)	4 (2.44%)	3 (1.88%)

Note:- Denominators are subjects who experienced side effects

## Discussion

This survey was conducted to assess trachoma post MDA coverage in East Wollega, Horo Guduru Wollega, and West Shewa Zones of Oromia. In this survey, four of the six districts met the 80% target threshold for effective coverage recommended by WHO [11]. For Ambo Rural and Jimma Geneti districts, the survey coverage was less than the target coverage threshold. When extrapolating to zonal level, the two districts of East Wollega zone showed better coverage compared to West ShewaShewa and Horo Guduru Wollega zones.

While it was found that the majority of surveyed HHs (73.4%) had received information about trachoma, this figure was lower than in Injibara Town and adjacent Banja district of Awi Zone, Amhara Region, where 94.9% of HHs had ever heard about trachoma [12]. The discrepancy between the studies might be due to the reason that the current study was conducted in rural districts where information access is limited. However, the figure reported here is higher than in Kenya among pastoralist patients in Kajiado Central Division where 65.7% and 64.1% of the respondents had heard about trachoma among both child caretakers and the adult trachoma respondents [13]. Health workers were the major source of information for trachoma and MDA in all surveyed districts. A similar finding was found in Injibar, Mojo, and Lume Injibar [12, 14].

Fear of side-effects, absence during the MDA campaign, drug distributor no-shows and under age status were the mentioned reason for not swallowing the drug—these are similar findings to a study conducted previously in Amhara and Tigray regional states [12, 15]. Another study conducted in Kenya also showed similar results, in which, lack of awareness, fear of side-effects, migration and grazing animals were reported as barriers to swallowing medication [16]. Compared to a previous survey conducted in Ethiopia in which approximately 70% of eligible persons in the surveyed HHs received their allocated dose of azithromycin at each time point; the MDA coverage in this study area was seen to be higher as it was at least 80.5% [17]. This could have resulted from the difference in time of the study.

In this survey, in 2/3 of the total surveyed districts, more males than females swallowed drugs. Similarly, children aged 5–14 and 0–4 years were the largest and smallest age group swallowing drugs, respectively. The participation of children aged 1–9 years, the target age group of the trachoma control program, in the MDA was 91.42%; this was much higher when compared to a coverage survey conducted in Nigeria, Plateau state, where participation was 58.8% [18]. Thus, in this survey area, the last MDA campaign reached almost all of the target group.

### Limitations of the survey

There were problems of accessibility due to road and bridge damage resulted from rainy season. As a result, some kebeles were replaced by other accessible kebeles with a similar setup. This might have either overestimated or underestimated the coverage results which may affect the representativeness of the survey. Dependency of the data on self-reporting; recall bias; desirability bias; and only getting people who are home during the time of data collection were also limitation of the study. House hold level responses might have also not represented the other family members.

### Conclusion

In this survey, four of the six districts met the 80% target threshold for effective coverage; Ambo Rural and Jimma Geneti districts did not meet the target threshold indicating that programmatic improvements will have to be developed and applied in future MDA rounds. MDA coverage observed through the current survey was lower than reported coverage, except for Haro Limu and Boneya Boshe districts. While the difference was small (2%) in Haro Limu it was high (20%) in Boneya Boshe; further investigation is needed to identify why such high discrepancy was recorded between reported and surveyed coverage in the latter district. The majority of HHs in this survey got information about trachoma and MDA from health workers followed by radio; other sources of information like public announcements should also be encouraged to reach the whole population since a significant number of the HHs surveyed stated that they did not hear about the last MDA campaign.

## Supporting information

S1 Dataset Individual level result-data in SPSS format. (SAV)

S2 Dataset Family level result-data in SPSS format. (SAV)
